# A think-aloud study of the feasibility of patients with end-stage organ failure completing the ICECAP-SCM

**DOI:** 10.1177/02692163221122979

**Published:** 2022-09-16

**Authors:** Henry Nwankwo, Joanna Coast, Alistair Hewison, Philip Kinghorn, Shyam Madathil, Cara Bailey

**Affiliations:** 1 Centre for Health Economics at Warwick, Clinical Trials Unit, University of Warwick, Coventry, UK; 2 Health Economics Bristol, Population Health Sciences, Bristol Medical School, University of Bristol, Bristol, UK; 3 School of Nursing, Institute of Clinical Sciences, University of Birmingham, Birmingham, UK; 4 Health Economics Unit, Institute of Applied Health Research, University of Birmingham, Birmingham, UK; 5 Respiratory Medicine Department, University Hospitals Birmingham NHS Foundation Trust, Birmingham, UK

**Keywords:** Palliative care, end-of-life care, economic evaluation, quality of life, measurement, heart failure, COPD, ESRD

## Abstract

**Background:**

The ICECAP-Supportive Care Measure (SCM) is a self-complete measure developed to inform economic decision making at the end-of-life. Previous research has demonstrated its feasibility in hospice and nursing home settings. This is the first study of its use with patients on the organ failure trajectory.

**Aim:**

To determine the feasibility of using the ICECAP-SCM with patients experiencing end-stage organ failure in a hospital setting.

**Design:**

Participants were asked to ‘think aloud’ when completing the ICECAP-SCM, ICECAP-A and EQ-5D-5L measures. The interviews were transcribed verbatim and examined for errors in comprehension, retrieval, judgement, and response by five raters. Qualitative data were collected to explore reasons for errors in completing the measures and participants’ views about the measures.

**Setting/participants:**

Sixty patients (with end-stage renal failure *n* = 18; end-stage heart failure *n* = 21; end-stage chronic obstructive pulmonary disease *n* = 21) participated. Senior clinicians applied prognostic criteria to determine eligibility.

**Results:**

Participants reported that the measures were acceptable, clear, and easy to complete. Error rates in completing the measures were low (ICECAP-A = 3%,and ICECAP-SCM = 5.7% and EQ-5D-5L = 6.3%). There was some variation in responses between patients with different end-stage conditions, particularly those with symptom fluctuation. Some patients had not considered their end-of-life (i.e. advance care planning) and reported finding questions about this difficult to answer.

**Conclusion:**

It is feasible to use the ICECAP-SCM with patients with end-stage organ failure receiving care in hospital settings. This study provides evidence for researchers and policy makers involved in measuring end-of-life care globally. The ICECAP-SCM can be recommended for research with patients in end-stage organ failure to appropriately capture the broader benefits of end-of-life care.

**What is already known about this topic?**

- Generic economic measures used to evaluate the benefits of end-of-life care interventions have been criticised as being inappropriate and too narrowly focused to capture the broader benefits of end-of-life care interventions.
- The ICECAP-Supportive Care Measure was developed to measure capability wellbeing at the end of life, for use in economic evaluation
- The ICECAP-SCM has been investigated and shown to be feasible in patients receiving care in hospice and nursing home settings. This is the first study to investigate the feasibility of using the ICECAP-SCM in patients on the organ failure trajectory in a hospital setting.

**What this paper adds?**

- The study demonstrates that the ICECAP-SCM is feasible to use in evaluating health and wellbeing in patients with end-stage organ failure in a hospital setting.
- Questions focussing on end-of-life decision making and capability wellbeing at the end of life can be emotionally difficult for patients to complete.

**Implications for research, theory, or policy**

- The ICECAP-SCM can be used alongside the EQ-5D-5L and ICECAP-A (a measure of generic well-being for adults) in an integrated framework to inform economic decision making in patients at different stages on the end stage organ failure trajectory.
- The ICECAP-SCM addresses methodological issues in measuring quality of life in patients with fluctuating symptoms. It offers the opportunity for a more appropriate estimation of benefits in patients on the organ failure trajectory who experience fluctuating symptoms.

## Introduction

An ageing population and the rising cost of end-of-life care interventions and services have increased the scrutiny of researchers and policy makers on decision making processes at the end-of-life.^1,2^ Globally, about 40 million people are estimated to need end of life care annually with significant economic costs.^3^ In many countries, decision makers use frameworks that employ a range of implicit and explicit criteria to guide resource allocation decisions in health care.^4^ In countries with health systems funded by direct taxation, public institutions such as the National Institute of Health and Care Excellence (NICE) in the United Kingdom, Pharmaceutical Benefits Advisory Committee (PBAC) in Australia and the Canadian Agency for Drugs and Technologies in Health (CADTH); are tasked with improving the process of allocating health care resources.^5^ In common with a number of other countries, NICE relies in part on the cost-effectiveness of health interventions to guide resource allocation decisions.^5^ Cost utility analysis is recommended by NICE to appraise the economic benefits of health interventions^6^ by comparing two or more interventions in terms of their costs and outcomes.^7^ Outcomes are generally expressed as Quality-Adjusted Life-Years (QALYs), a measure of health outcome where life following a health intervention is adjusted for quality.^7^ Generic preference-based outcome measures are used to assess the cost-effectiveness of health interventions, with the EQ-5D-5L most commonly advised for this purpose.^8^

The use of QALYs in evaluating end-of-life care and economic decision making at the end-of-life has been criticised on the basis that end-of-life interventions are not exclusively aimed at improving health-related functioning, they also focus on providing care, managing symptoms, and achieving a good death^9–11^ Researchers have suggested that a broader focus for decision making than one centred on health-related functioning, may provide a more appropriate basis for evaluating end-of-life care.^12^ The capability approach normatively distinguishes between functionings and capabilities. Functionings are defined as ‘beings’ and ‘doings’ for example being in good health, walking, being well nourished, while capabilities refers to the freedom of an individual to ‘do’ and ‘be’ what they have reason to value.^13^ The capability approach offers an alternative theoretical framework that considers the extent that a person is able to do and be the things they have reason to value, as a basis for evaluation.^14^

The ICECAP-Supportive Care Measure (ICECAP-SCM) was developed as a self-complete economic measure based on the capability approach to evaluate health and wellbeing associated with the opportunity for a good end-of-life.^15^ The feasibility of using the ICECAP-SCM with patients receiving palliative care in hospice and nursing home settings has been demonstrated.^16,17^ However, there remain questions about its feasibility for use with patients on different dying trajectories^18^ and in different care settings. This is important because hospice patients may have different experiences and needs to hospital inpatients or those not accessing, or not able to access, specialist palliative or supportive care services.

Whilst a single measure is commonly used for evaluating the benefits of health interventions, the multi-faceted objectives of palliative care have led to a growing recognition that a single outcome might not suffice at this stage of the life-course.^19^ Improving health, managing symptoms and achieving a good death take on varying degrees of prominence at various points along the dying trajectory.^20^ It is essential that current evaluative methods account for these shifting objectives in economic decision making.

The aim of the study reported here was to investigate the feasibility of using the ICECAP-SCM, alongside more established economic measures focused on health (EQ-5D-5L) and generic capability (ICECAP-A),^21^ with patients on an organ failure trajectory receiving treatment and care in a hospital setting. It was designed to address the following research questions: what difficulties did participants face while completing the ICECAP-SCM alongside other economic measures? What are the views of participants about these measures, in relation to their health and wellbeing?

## Methods

Think-aloud interviews involving retrospective verbal probing, followed by a semi-structured interview were used to explore patients’ perceptions of end-of-life care and the feasibility of using the ICECAP-SCM, EQ-5D-5L and ICECAP-A measures. The think-aloud interview is a form of cognitive interview in which respondents are asked to verbalise their thoughts as they complete each measure.^22^ Think-aloud interviews have been effective in gaining deeper insight into decision making and experiences of people at the end-of-life.^23^ Responses were probed retrospectively to clarify any difficulties experienced in completing the measures during the think aloud interviews to help ensure the rigour and trustworthiness of the data. The research was granted ethical approval from the North Wales Research Ethics Commission and the Health Research Authority (REC reference: 17/WA/0022).

## Sampling and recruitment

There are no specific recommendations for sample size in think-aloud studies and numbers of participants have ranged from as few as 9^24^ to as many as 72.^16^ The aim in the present study was to recruit a total of 60 patients including at least 20 with each of end-stage heart failure (HF), end-stage chronic obstructive pulmonary disease (COPD) and end-stage renal disease (ESRD). It was anticipated that a sample of 60 patients would be sufficient to enable comparability of scores for the measures and achieve data saturation for the analysis of verbal responses.^25,26^

People near the end-of-life were defined as those in the last 12 months of life.^27^ There are acknowledged difficulties in identifying patients with end-stage organ failure.^28,29^ In view of this prognostic criteria were developed in consultation with specialists in the renal, respiratory and HF units to increase the likelihood that patients in the last 12 months of life were recruited (Appendix 2).

Recruitment teams of a consultant and at least one specialist nurse were set up in the HF and respiratory units, led by SM. The recruitment teams were responsible for identifying patients eligible for inclusion based on condition-specific prognostic criteria (Appendix 2). Patients were screened (by the recruitment team) against the prognostic criteria and those deemed eligible for inclusion eligible were then screened (by HN) against the generic eligibility criteria (Appendix 3). Patients meeting both sets of criteria were invited to participate in the research. Patients with ESRD who were included had declined renal replacement therapy and dialysis in favour of palliative care. They were recruited from a specialist renal out-patient palliative clinic by the hospital renal team. Patients with end-stage HF and end-stage COPD were purposively recruited from in-patient wards.

Following initial contact by the consultant in charge of their care, potential participants were provided with an information sheet by HN, containing the details of the study. Whilst there was no formal patient and public involvement in the study, the Patient Advisory and Liaison Service of the hospital provided feedback on the research design and topic guide.

### Measures investigated

The ICECAP-SCM is a measure of capability well-being at the end-of-life.^15^ It consists of seven attributes: choice (being able to have a say), love (being able to be with people who care about you), freedom from physical suffering, freedom from emotional suffering, dignity (being able to maintain one’s dignity), support (being able to have support), and preparation (having the opportunity to make end-of-life decisions). Each attribute is expressed at four levels of capability and their wish for that capability; ranging from no capability to full capability. Respondents are asked about their wellbeing ‘at the moment’. The ICECAP-SCM has been tested for feasibility with patients receiving care in a hospice^16^ and as a proxy instrument for people living with advanced dementia in nursing homes^17^ and tariffs have been developed following valuation exercises in samples derived from UK^30^ and German adult populations.^31^

The ICECAP-A is a measure of the generic capability of adults, and asks respondents to rate their quality of life ‘at the moment’.^21^ It consists of five attributes (Stability, Attachment, Autonomy, Achievement and Enjoyment), expressed as four levels of capability ranging from no capability to full capability. The ICECAP-A has been subject to extensive validity testing with different groups of patients,^16,32,33^ including patients with mental health problems.^34^

The EQ-5D-5L is a measure of health functioning recommended for use in economic evaluation of health and social care interventions in the UK.^6^ It has five attributes (Mobility, Self-Care, Usual activities, Pain/Discomfort, Anxiety/Depression) expressed at five levels of severity ranging from no problems to extreme problems.^8^ The EQ-5D-5L has been used to evaluate end-of-life interventions and services.^35,36^ It measures health status in terms of health gains or loss and asks respondents to rate their quality of life ‘today’.

### Data collection

Think-aloud face-to-face interviews were conducted at the hospital site or the patient’s home (dependent on participant preference). All interviews were conducted by HN. Prior to each interview, participants were asked to perform a think-aloud exercise to familiarise them with the think-aloud process. Following the warm-up task, involving them speaking about the number of encounters they had with health professionals on an admission to hospital, participants were asked to confirm their age, race, and gender. Participants were then given the measures in a random order and encouraged to verbalise their thoughts (‘think aloud’) as they made their response to each item. If participants were silent for longer than 10 s, they were prompted to keep thinking out loud. Following completion of all three measures, respondents were asked additional ‘probe’ questions to explore any difficulties the participants experienced and clarify any unclear responses.

End of life is a sensitive topic, and two participants became upset during the interview. When this occurred, the issue was handled sensitively, and participants were asked whether they would like to stop or continue with the interview. A distress protocol was also developed to ensure such episodes were managed appropriately (see p.12).

### Data analysis

All interviews were audio-recorded and transcribed verbatim. Think-aloud responses to the items in each measure and their scores were analysed by five independent raters (H.N., J.C., P.K., A.H. and C.B.) to increase rigour. Each item was rated to identify if any of four types of response problems^37^ were evident: comprehension (any misunderstanding of a word or phrase), retrieval (a recall error or miscalculation of the time frame stated in the question), judgement (correctly judging how recalled information can be used to answer the question), and response (providing a valid response to the question answered). Additionally, a fifth category, ‘struggle’ (not an error) was included as in previous ICECAP ‘think aloud’ studies; this was used to capture instances when participants arrived at an appropriate response that reflected their discussion but clearly (from their discussion) found it difficult to get to this response.^16,38,39^ Examples of errors and ‘struggle’ can be found in the supplementary file (Appendix 1).

An error/struggle classification was made when three or more raters identified a specific error/struggle type in an item response. In cases where three or more raters identified an issue but disagreed on the category, discussion took place to reach a consensus on the specific error/struggle type. When two raters identified an error/struggle, this was discussed in the group to reach a consensus. If only one rater identified an error/struggle, it was not classified as an error/struggle. Error/struggle rates were calculated as percentages of the number of items completed to enable comparability as the ICECAP-SCM contains more questions than the other measures.

Transcripts of the think-aloud interviews were read and re-read, and codes were generated inductively through open coding.^40^ Data were compared across transcripts and to the properties of emerging categories. Qualitative analytic accounts^41^ were written for batches of transcripts (6–10 patients with each condition) to enable a rigorous process for assessing the reasons for errors and participants’ views about the measures. Qualitative data were managed in NVivo© 12.1 software by H.N. Each stage of the analytic process was reviewed by C.B, J.C., A.H. and P.K.

## Results

One hundred and sixteen patients were identified as eligible for inclusion based on the prognostic criteria. They included 47 patients with ESRD, 41 with end-stage COPD and 28 with end-stage heart failure. Nineteen patients were excluded by the recruitment team for the following reasons: three patients were too ill to be interviewed; one patient was discharged; one died before an interview could be arranged; six had cognitive issues and were unable to understand the information sheet; and eight could not communicate in the English language. Ninety-seven patients were invited to participate in the research and 37 declined. The reasons given for declining participation are presented in Table 1.

**Table 1.** Reasons given by eligible patients for their decision not to participate in the study.

Reasons for decline	ESRD	COPD	HF	Total
Breathing difficulties	0	5	0	5
Poor eye sight	1	1	0	2
Upsetting speaking about quality of life.	1	2	0	3
Transportation issues	4	0	0	4
Non-consent to interview/audio record	1	4	1	6
Pain	0	1	0	1
Bereavement	0	1	0	1
Fatigue	0	0	1	1
Unease with research	1	0	0	1
No reason given	9	3	1	13
**Total**	**17**	**17**	**3**	**37**

Participants were recruited at various stages of their illness trajectory including those who were very near the end-of-life; 23 of the 60 participants died within 8 months following the interview including 19 who died within 4 months of interview. Interviews were conducted between June and November 2017. Patients included in the study were aged between 35 and 95 years. Interview duration ranged from 14 to 49 min. The socio-demographic characteristics of the participants are presented in Table 2.

**Table 2.** Socio-demographic information of participants.

Characteristics	ESRD *n* = 18	COPD *n* = 21	HF *n* = 21	Total *n* = 60
Age Group (years)				
<60	0 (0%)	3 (14.3%)	3 (14.3%)	6 (10%)
60–79	2 (11.1%)	12 (57.1%)	10 (47.6%)	24 (40%)
>80	16 (88.9%)	6 (28.6%)	8 (38.1%)	30 (50%)
Gender				
Male	11 (61.11%)	6 (28.57%)	15 (71.43%)	32 (53%)
Female	7 (38.89%)	15 (71.43%)	6 (28.57%)	28 (47%)
Race				
White	14	20	20	54 (90%)
Black	3	1	1	4 (8%)
Asian	1	0	0	1 (2%)
Interview location				
Home-based interview	4	0	1	5 (8%)
Hospital-based interview	14	21	20	55 (92%)

### Completion of the measures

Thirty-four of the 60 participants completed all three measures without errors, although six of these experienced struggle. The lowest error rate (3%) was demonstrated in completion of the ICECAP-A which 52 of the 60 participants completed without error. The error rate for completion of the EQ-5D-5L was the highest at 6.3%, compared with 5.7% for the ICECAP-SCM. The EQ-5D-5L and ICECAP-SCM were each completed by 45 of the 60 participants with no errors.

Participants with ESRD had the highest percentage error rate when completing the ICECAP-SCM (8.7%) and the lowest percentage error rate when completing the EQ-5D-5L (2.2%); while those with end-stage heart failure and end-stage COPD had the highest percentage error rate completing the EQ-5D-5L (9.5% and 6.7%, respectively). Absolute and percentage error rates for the three patient groups are shown in Table 3.

**Table 3.** Errors, struggle and percentage error rates in completion of the measures.

	ESRD [*N* = 18]	COPD [*N* = 21]	HF [*N* = 21]	Total [*N* = 60]
EQ-5D-5L				
°Errors (P)	2 (11%)	6 (29%)	6 (29%)	14 (23%)
°Struggle (P)	1 (6%)	3 (14%)	2 (10%)	6 (10%)
°No error/struggle (P)	15 (83)	12 (57%)	13 (62%)	39 (65%)
°Error (n)	2 (2.2%)	7 (6.7%)	10 (9.5%)	19(6.3%)
°Struggle (n)	1 (1.1%)	3 (2.9%)	2(1.9%)	6(1.7%)
ICECAP-A				
°Errors (P)	1 (6%)	2 (10%)	5 (24%)	8 (13%)
°Struggle (P)	1 (6%)	1 (5%)	0	2 (3%)
°No error/struggle (P)	16 (89%)	18 (86%)	16 (76%)	50 (83%)
°Error (n)	1(1.1%)	3 (2.9%)	5 (4.8%)	9(3%)
°Struggle (n)	1 (1.1%)	1 (1%)	1 (1%)	3(1%)
ICECAP-SCM				
°Errors (P)	5 (28%)	6 (29%)	4 (19%)	15 (25%)
°Struggle (P)	2 (11%)	0	0	2 (3%)
°No error/struggle (P)	11 (61%)	15 (71%)	17 (81%)	43 (72%)
°Error (n)	11 (8.7%)	7 (4.8%)	6 (4.1%)	24 (5.7%)
°Struggle (n)	2 (1.6%)	1 (0.7%)	0	3 (0.7%)
Total				
°Error (P)	7 (39%)	10 (47.6%)	9 (42.9%)	26 (43.3%)
°Struggle (P)	2 (11%)	2 (10%)	2 (10%)	6 (10%)
°No error/struggle (P)	9 (50%)	9 (43%)	10 (48%)	28 (47%)
°Error (n)	14 (4.6%)	17(4.8%)	21 (5.9%)	52(5.1%)
°Struggle (n)	4 (1.3%)	5 (1.4%)	2 (0.6%)	12(1.2%)

P: Number of participants with/without errors or struggle.

n: Number of errors or struggle.

N: number of participants in a group.

Participants completing the ICECAP-SCM made the most errors when completing the ‘preparation’ and ‘emotional suffering’ items, as shown in Table 4. Comprehension and response errors were the most common type. Data extracts are included below to illustrate such errors. Most errors in ‘preparation’ were comprehension errors, with some participants appearing to conflate preparation with day-to-day decision making rather than end-of-life decision making.

“I can make my own meal. I’ve got some frozen food in the freezer. I can do those. My daughter makes some food and keeps them in a box and I can do them” [PT-54R]

“I can dress myself, I can wash myself, I don’t need anybody to give me baths, so far I’m ok on the food side” [PT-18K]

**Table 4.** Nature of errors across attributes of the ICECAP-SCM among participant groups.

Error	Choice	Love	Physical Suffering	Emotional suffering	Dignity	Support	Preparation	total
Comprehension								
°ESRD	0	1	0	1	0	0	3	**5**
°COPD	0	0	1	0	0	0	2	**3**
°HF	0	0	0	1	0	0	1	**2**
Retrieval								
°ESRD	0	0	0	0	0	0	0	**0**
°COPD	0	0	0	0	0	0	0	**0**
°HF	0	0	0	0	0	0	0	**0**
Judgement								
°ESRD	0	0	0	0	0	0	0	**0**
°COPD	0	0	0	0	0	0	0	**0**
°HF	0	0	0	0	0	0	0	**0**
Response								
°ESRD	0	0	2	3	0	1	0	**6**
°COPD	0	0	0	1	0	1	2	**4**
°HF	1	0	0	0	1	1	1	**4**
Total	**1**	**1**	**3**	**6**	**1**	**3**	**9**	**24**

Similarly some participants explained how they were able to make independent decisions in general terms, rather than focussing on preparation for the end-of-life.

“I make a decision myself. I have to ask somebody if I want to make a decision very rare now. I make my own decisions”. [PT-63R]

Some participants made response errors when completing the preparation item. In most cases, the question was left unanswered. When asked why the question was unanswered, it was because they had not considered or made specific preparations.

“I have my financial affairs in order. . .I don’t know, I don’t know about that question. . .

no, no, no, I don’t think so . . . I don’t know about that one” [PT-33 R]

Although participants made the fewest errors completing the ICECAP-A, most errors were made in response to the question about ‘achievement and progress’. Participants questioned the relevance of ‘progress’ to their wellbeing.

“ I don’t know what that means really. ‘Progress in many aspects of my life’. What would that be? Achievements? I don’t understand that” [PT-48K]

Participants did not make any comprehension errors while completing the EQ-5D-5L. Most errors were response errors and were due to the description of the response options. Some participants felt unable to make a selection from the options available when they felt that the item described two different dimensions. For example, the EQ-5D-5L includes a question about anxiety and depression and if people had experienced one but not the other they were sometimes unable or unwilling to answer.

“I think they needed to be worded differently. . . like with that one there, anxiety and depression I think it should have like, am I slightly anxious, yes but do I feel a bit depressed? I know anxiety can lead to depression but a lot of people suffer with anxiety for years and years but they don’t become depressed. Like myself, I’ve had you know, I’ve been anxious over time but I’m not depressed” [PT-24H]

Many participants felt the severity of their condition fluctuated on a day-to-day basis which made it difficult to quantify their wellbeing. This was common among those with end-stage HF and end-stage COPD who did not feel the options in the EQ-5D-5L (expressed in terms of severity) described their condition.

“I could do both of those. I have moderate problems. . . and I have severe problems doing my usual activity. It’s all according to how I am on the day, so I don’t know” [PT-28R]

“See to me it could be one of any of them. . . You see that’s the problem if my legs are bad. . . it affects my breathing and my walking and everything. . . That’s when I come under two categories” [PT-59H]

The EQ-5D-5L appeared to be less sensitive to the frequent fluctuations of end-stage organ failure and so the participants did not feel the options they were offered to describe their condition accurately represented their current state.

### How did participants feel about completing the ICECAP-SCM?

Although a few participants struggled to complete some questions, most participants felt the ICECAP-SCM was clear and easy to complete.

“they were simple questions; they were straightforward to me” [PT-38K]

Indeed, many participants were able to articulate their responses in terms of their capabilities. While answering the questions, participants spoke about their wellbeing in terms of what they were willing and able to do.

“if you want to do it properly, you’ve got to think about what you want to do, what you might do and what you can’t do and that’s mainly the basis of it” [PT-29K]

Some participants however found reflecting on their capability wellbeing and the impact of their illness on family members upsetting.

“I do feel like a burden. I am a burden to them all. . . that’s upsetting and things I can’t do when I definitely want to do them” [PT-23R]

The think-aloud approach encouraged reflection and participants were often aware of their imminent death and became emotional while answering the question about ‘preparation’ in the ICECAP-SCM and the life they would soon leave behind. This was particularly common among participants in the late end-of-life phase (died within 4 months of interview)

“I understand how these professional footballers feel when their glittering career comes to an end and the crowd are completely gone. I don’t think I’m any different to anybody else on that, I think everyone is like that because nobody on this planet likes losing, nobody likes to go earlier than they should do. . . it’s gone ever so quick [cries]” [PT-45R Male, 76, died within 4 months of interview]

“I left my [children] with [relatives]. . . I’d love to say good bye. I know that I’m going to die, and I don’t want to die, I don’t want to leave here, sorry [cries]” [PT-37H, Female, 35, died within 4 months of interview]

Responding to the inevitability of death was challenging and painful, particularly when the participant felt that the end stage was reached quickly, leaving little time for them to prepare and plan for their death.

## Discussion

### Summary of findings

This research examined the feasibility of using the ICECAP-SCM, alongside the EQ-5D-5L and ICECAP-A with patients on an organ failure illness trajectory experiencing end-stage organ failure as they approached the end-of-life. Most participants felt the ICECAP-SCM was clear and easy to complete and it compared favourably with the other measures in terms of error rate, although the ICECAP-A had the lowest error rate.

Most errors made while completing the EQ-5D-5L were response errors and were due to difficulties experienced by participants with heart failure and COPD in quantifying their symptoms. Similarly, most errors in the ICECAP-SCM were in the ‘emotional suffering’ and ‘preparation’ items and these errors were due to difficulties in discussing advanced care planning and the impact of their illness on their emotional wellbeing. The ICECAP-A did not address sensitive topics and focuses on capability wellbeing rather than functioning hence most participants may have found it easier to complete.

The descriptive levels of the ICECAP-SCM, expressed in terms of frequency, appeared to reflect the wellbeing of participants more accurately, particularly those with end-stage HF and end-stage COPD who frequently experienced fluctuating symptoms. The focus of the ICECAP-SCM on capabilities associated with end-of-life decision making appeared to be difficult for some participants who became upset while reflecting on the impact of their condition on their emotional wellbeing and the inevitability of death. A distress protocol considering appropriate support mechanisms^42^ should be in place when using the ICECAP-SCM or any research that involves asking sensitive questions to people near the end of life.

### Relationship to wider literature

This is the first study to explore the feasibility of using the ICECAP-SCM in patients with end-stage organ failure. A previous study of the ICECAP-SCM in comparison to other measures in a nursing home setting reported a higher error rate for the ICECAP-SCM,^17^ while a study conducted in a hospice setting^16^ reported a lower error rate. Consistent with the findings reported here, Bailey et al. noted that participants had difficulties discussing advanced care planning, reflected in more errors in responding to the ‘preparation’ items.^16^ Despite the challenges involved in having discussions with people near the end-of-life,^43^ they are generally seen as beneficial to patients^44^ and can lead to more efficient use of health care resources.^45^

The organ failure trajectory, with its inherent rapidly fluctuating health states, makes measurement of health functioning particularly challenging and use of existing measures can lead to inaccurate estimates of the benefits of interventions.^20,46^ The descriptive levels of the EQ-5D-5L, expressed in terms of severity, made it difficult for participants to quantify their health state due to fluctuations in their condition, as found in other studies.^16,47^ This problem of fluctuating health states is not unique to end-of-life, and is the subject of ongoing research^46^ The descriptive levels of the ICECAP-SCM expressed in terms of frequency may be more appropriate in evaluating end-of-life care outcomes in those very near the end of life.

### Implications for research and policy

Despite the challenges involved in using the measures for patients with end-stage organ failure, all three measures were feasible to use and accepted by patients. The holistic approach to care advocated in national and international end of life care policy^48,49^ underscores the need for a range of measures to capture the diverse benefits of interventions across the end-of-life course. The ICECAP-SCM has been translated in five different languages which will facilitate its use in capturing outcomes beyond health functioning in low- and middle-income and non-English speaking countries. Health, generic capability, and capabilities associated with end-of-life decision making take on varying degrees of relative importance as patients move through the end-of-life course.^50^ Using all three measures in an integrated framework may provide a more accurate approach for evaluating the impact of palliative care interventions in end-of-life research for patients on the organ failure trajectory.

### Strengths and limitations

The study has several strengths and some limitations. Sixty participants with end-stage organ failure participated in the research, making it the largest patient-completed think-aloud study using any of the ICECAP measures to date. The research was successful in recruiting people who were very near the end-of-life; a challenging group to recruit. The accuracy of the application of the prognostic criteria in selecting participants for the study is confirmed by the fact that 23 of 60 participants died within 8 months of being interviewed. Some participants died within a few days of being interviewed, demonstrating that people at different stages of the organ failure trajectory were included. Furthermore, participants were purposively recruited with different conditions causing end-stage organ failure who received different forms of care ensuring the generalisability of findings given contemporary evolution in palliative care delivery models. The study has some limitations. All participants in the research were recruited from one geographical location, in a single site in England and most self-identified as white-British. There is however no evidence of regional and ethnic differences in end of life patients completing the ICECAP measures. Previous studies^16,17^ using similar methods recruited from a single site with participants mainly identifying as white British. Hence, future studies using the ICECAP-SCM would benefit from being repeated with different ethnic groups in a range of geographical locations to explore its use across a more diverse population and identify any potentially challenging aspects of measuring quality of life. Given the need for a range of measures to evaluate the benefits of interventions across end-of-life trajectories, further research on how best to combine the measures and the challenges and feasibility of doing so would be beneficial.

## Conclusion

The ICECAP-SCM was specifically developed to evaluate health and capability wellbeing at the end-of-life and this study has shown that use of the measure is feasible and acceptable to patients with end-stage organ failure. Used in conjunction with the EQ-5D-5L and ICECAP-A, it provides researchers and policy makers with an approach to evaluate the impact of end-of-life interventions and provides a more appropriate estimation of the benefits of end-of-life care.

## Appendix 1. Examples of each error type.

**Table:**

	Error	Not error
Comprehension	I want to be able to – people who care about me some of the time. Well, I know my son cares about me. I want to be able to be with people who care about me only a little of the time. No, I don’t want - I want the answer to be able to be with people who care about me. I don’t understand half of them, really.	Love? I’m not sure what that means. I will assume it’s about having my family around. [Box ticked]
Retrieval	Love? Well, I had a lot of love a few years ago. It’s all changed now, but I think I will go with ‘a lot of love’.	Love? Well, I had a lot of love a few years ago, it’s hard to say now, but I think I will go for that box.
Judgement	Love? Well it’s asking about how much love I can have, which isn’t much. . . from people anyway. . . but I love lots of things, I love going to the cinema, so I will say ‘a lot of love’.	Love? Well I love lots of things and can get lots of love. I love going to the cinema. . . I will say ‘a lot of love’.
Response	No. I often experience physical discomfort but it’s just my legs and my back and if I have a paracetamol and then it eases off, you see, and I can walk. I can walk without my four-wheeler or anything but it’s not discomfort, it isn’t. [ticked full capability in relation to physical discomfort]	Well I would say I can have some love and friendship. . .I guess that’s closest to ‘quite a lot’, so I will tick that box [Box ticked] Well I would normally say I can have a lot of love and friendship, because you wouldn’t normally want to own up to not being able to [response to measure is NOT ‘a lot of love and friendship’]]
Struggle	Love? It’s just such a difficult concept. . .can I have a lot? What does ‘can’ mean – I don’t know! I’m just going to go with this answer, but I find it a confusing question. [Answer ticked]	Love? It’s a difficult concept. . .can I have a lot? What does ‘can’ mean? I can have support in my life that’s fine. [Answer ticked]

## Appendix 2

### Prognostic criteria

#### End-stage heart failure

1. History of severe heart failure at baseline (New York Heart Association Class III or IV) despite being treated medically with appropriate drugs as by their clinician;
2. A definite history of New York Heart Association Class IV CHF at hospital admission or ICU transfer as manifested by baseline dyspnoea at rest related to primary cardiac failure, systolic blood pressure 100 mm Hg or less related to primary cardiac failure, or a history of hypotension despite the use of recommended medications;
3. Chart documentation of CHF and a left ventricular ejection fraction less than or equal to 35%;
4. QRS with left bundle branch block configuration, and duration >120 ms.

#### End Stage Renal Disease

1. Glomerular filtration rate of < 15 mL/min and/or
2. Diagnosed as ESRD* (i.e. eligible for dialysis in the anticipation that they will need renal replacement therapy or an indefinite haemodialysis) and
3. Managed by conservative care i.e. patients who decline renal replacement therapy or dialysis.

Patients may or may not have had dialysis in the past but patients participating in the study were expected to be on conservative therapy.

#### End-stage COPD

Patients suspected of having end stage COPD were expected to have at least two of the indicators below:

1. Disease assessed to be severe (e.g. FEV1 < 30% predicted)
2. Recurrent hospital admissions (at least 3 in last 12 months due to COPD)
3. Fulfils long term oxygen therapy criteria
4. MRC grade four-fifths – shortness of breath after 100 m on the level of confined to house
5. Signs and symptoms of right heart failure
6. Combination of other factors, that is, anorexia, previous Intensive Therapy Unit/Non-Invasive Ventilation resistant organisms.
7. More than 6 weeks of systemic steroids for COPD in preceding 6 months

## Appendix 3

### Eligibility criteria

Participants were expected to meet the following generic eligibility criteria:

1. Participants were able to provide informed consent.
2. Participants were willing and able to directly participate
3. Participants able to communicate in the English language
4. Participants were over 18 years of age.

Patients who are not be able to give consent or directly participate in the study will be excluded from the study.

## References

[1] Cylus J , et al. Will population ageing spell the end of the welfare state? A review of evidence and policy options. Geneva: World Health Organization, 2019, Copenhagen: Denmark. 31820887

[2] Townsend M. The impact of an ageing population on end of life care costs. London: London School of Economics, 2016.

[3] WHO. Palliative care: Key facts. https://www.who.int/news-room/fact-sheets/detail/palliative-care (2020, accessed 25 March 2022).

[4] Seixas BV Regier DA Bryan S , et al. Describing practices of priority setting and resource allocation in publicly funded health care systems of high-income countries. BMC Health Serv Res 2021; 21(1): 90. 33499854 10.1186/s12913-021-06078-z PMC7839200

[5] Whyte P Hall C. The role of health technology assessment in medicine pricing and reimbursement. In: WHO/HAI Project on Medicine Prices and Availability. World Health Organization, 2013.

[6] National Institute for Health and Care Excellence. NICE process and methods guides, in guide to the methods of technology appraisal 2013. In: 2013, National Institute for Health and Care Excellence (NICE), London.

[7] Drummond MF , et al. Methods for the economic evaluation of health care programmes. New York: Oxford university press, 2015.

[8] Herdman M Gudex C Lloyd A , et al. Development and preliminary testing of the new five-level version of EQ-5D (EQ-5D-5L). Qual Life Res 2011; 20(10): 1727–1736. 21479777 10.1007/s11136-011-9903-x PMC3220807

[9] Hodiamont F Jünger S Leidl R , et al. Understanding complexity – the palliative care situation as a complex adaptive system. BMC Health Serv Res 2019; 19(1): 157. 30866912 10.1186/s12913-019-3961-0 PMC6417077

[10] Higginson IJ Evans CJ Grande G , et al. Evaluating complex interventions in end of life care: the MORECare statement on good practice generated by a synthesis of transparent expert consultations and systematic reviews. BMC Med 2013; 11(1): 111. 23618406 10.1186/1741-7015-11-111 PMC3635872

[11] Normand C. Setting priorities in and for end-of-life care: challenges in the application of economic evaluation. Health Econ Policy Law 2012; 7: 431–439. 23079301 10.1017/S1744133112000229

[12] Coast J. Strategies for the economic evaluation of end-of-life care: making a case for the capability approach. Expert Rev Pharmacoecon Outcomes Res 2014; 14(4): 473–482. 24784902 10.1586/14737167.2014.914436

[13] Sen A . The economics of happiness and capability. In Bruni Luigino Comim Flavio Pugno Maurizio (eds.), Capabilities and Happiness. Oxford University Press, 2008, pp. 16–27.

[14] Sen A . Equality of what? In: McMurrin S (ed.), Tanner lectures on human values, Vol. 1. Cambridge: Cambridge University Press, 1980, pp.197–220. https://scholar.harvard.edu/sen/publications/equality-what

[15] Sutton EJ Coast J. Development of a supportive care measure for economic evaluation of end-of-life care using qualitative methods. Palliat Med 2014; 28(2): 151–157. 23698452 10.1177/0269216313489368

[16] Bailey C Kinghorn P Orlando R , et al. The ICECAP-SCM tells you more about what I’m going through’: A think-aloud study measuring quality of life among patients receiving supportive and palliative care. Palliat Med 2016; 30(7): 642–652. 26819326 10.1177/0269216315624890 PMC4931711

[17] Froggatt K Best A Bunn F , et al. A group intervention to improve quality of life for people with advanced dementia living in care homes: the Namaste feasibility cluster RCT. Health Technol Assess 2020; 24(6): 1–140. 10.3310/hta24060 PMC7008353 31971506

[18] Lunney JR Lynn J Foley DJ , et al. Patterns of functional decline at the end of life. JAMA 2003; 289(18): 2387–2392. 12746362 10.1001/jama.289.18.2387

[19] French T Raman S Jindal-Snape D . Future transitions in palliative care: care across the life course for people with life-limiting conditions. Scottish Universities Insight Institute. https://www.scottishinsight.ac.uk/Programmes/Scotland2030/PalliativeCare.aspx (2019) (accessed November 2020).

[20] Murray SA Kendall M Boyd K , et al. Illness trajectories and palliative care. BMJ 2005; 330(7498): 1007–1011. 15860828 10.1136/bmj.330.7498.1007 PMC557152

[21] Al-Janabi H Flynn TN Coast J. Development of a self-report measure of capability wellbeing for adults: the ICECAP-A. Qual Life Res 2012; 21(1): 167–176. 21598064 10.1007/s11136-011-9927-2 PMC3254872

[22] Beatty PC Willis GB. Research synthesis: the practice of cognitive interviewing. Public Opin Q 2007; 71(2): 287–311.

[23] Bailey C Kinghorn P Orlando R , et al. Using think-aloud and interview data to explore patient and proxy completion of health and capability measures at the end of life. In Qualitative Methods for Health Economics. Rowman & Littlefield, 2017, pp. 231–244.

[24] Murtagh FE Addington-Hall JM Higginson IJ. The value of cognitive interviewing techniques in palliative care research. Palliat Med 2007; 21(2): 87–93. 17344256 10.1177/0269216306075367

[25] Aldiabat K Le Navenec C-L. Data saturation: the mysterious step in grounded theory method. Qual Rep 2018; 23(1): 245–261.

[26] Saunders B Sim J Kingstone T , et al. Saturation in qualitative research: exploring its conceptualization and operationalization. Qual Quant 2018; 52(4): 1893–1907. 29937585 10.1007/s11135-017-0574-8 PMC5993836

[27] General Medical Council. Treatment and care towards the end of life: good practice in decision making. General Medical Council, 2010.

[28] Cowie MR. The fine art of prognostication. Eur Heart J 2002; 23(23): 1804–1806. 12445530 10.1053/euhj.2002.3380

[29] Patel K Janssen DJ Curtis JR. Advance care planning in COPD. Respirology 2012; 17(1): 72–78. 22008225 10.1111/j.1440-1843.2011.02087.x

[30] Huynh E Coast J Rose J , et al. Values for the ICECAP-Supportive care measure (ICECAP-SCM) for use in economic evaluation at end of life. Soc Sci Med 2017; 189: 114–128. 28797940 10.1016/j.socscimed.2017.07.012

[31] Dams J Huynh E Riedel-Heller S , et al. German tariffs for the ICECAP-Supportive care measure (ICECAP-SCM) for use in economic evaluations at the end of life. Eur J Health Econ 2021; 22: 365–380. 33475868 10.1007/s10198-020-01260-2 PMC7954731

[32] Goranitis I Coast J Al-Janabi H , et al. The validity and responsiveness of the ICECAP-A capability-well-being measure in women with irritative lower urinary tract symptoms. Qual Life Res 2016; 25(8): 2063–2075. 26754141 10.1007/s11136-015-1225-y PMC4945699

[33] Keeley T Al-Janabi H Nicholls E , et al. A longitudinal assessment of the responsiveness of the ICECAP-A in a randomised controlled trial of a knee pain intervention. Qual Life Res 2015; 24(10): 2319–2331. 25894061 10.1007/s11136-015-0980-0 PMC4564441

[34] Mitchell PM Al-Janabi H Byford S , et al. Assessing the validity of the ICECAP-A capability measure for adults with depression. BMC Psychiatry 2017; 17(1): 46. 28148234 10.1186/s12888-017-1211-8 PMC5289054

[35] Mitchell PM Coast J Myring G , et al. Exploring the costs, consequences and efficiency of three types of palliative care day services in the UK: a pragmatic before-and-after descriptive cohort study. BMC Palliat Care 2020; 19(1): 119–9. 10.1186/s12904-020-00624-y PMC7412842 32767979

[36] Ciećko W , et al. Analysis of the quality of life of patients in the advanced phase of chronic diseases. Palliat Med Pract 2017; 11(2): 84–90.

[37] Tourangeau R Bradburn NM . The psychology of survey response. In: Handbook of survey research, 2nd ed. Cambridge: Cambridge University Press, 2010, pp.315–346.

[38] Al-Janabi H Keeley T Mitchell P , et al. Can capabilities be self-reported? A think aloud study. Soc Sci Med 2013; 87: 116–122. 23631786 10.1016/j.socscimed.2013.03.035 PMC3664929

[39] Mitchell PM Caskey FJ Scott J , et al. Response process validity of three patient reported outcome measures for people requiring kidney care: a think-aloud study using the EQ-5D-5L, ICECAP-A and ICECAP-O. BMJ Open 2020; 10(5): e034569. 10.1136/bmjopen-2019-034569 PMC7232621 32414822

[40] Miles MB Huberman AM Saldaña J. Qualitative data analysis: A methods sourcebook. London: SAGE publications, 2018.

[41] Coast J. Qualitative Methods for Health Economics. London: Rowman & Littlefield International, London, 2017.

[42] Draucker CB Martsolf DS Poole C. Developing distress protocols for research on sensitive topics. Arch Psychiatr Nurs 2009; 23(5): 343–350. 19766925 10.1016/j.apnu.2008.10.008

[43] Im J Mak S Upshur R , et al. “Whatever happens, happens” challenges of end-of-life communication from the perspective of older adults and family caregivers: a qualitative study. BMC Palliat Care 2019; 18(1): 113–119. 31830967 10.1186/s12904-019-0493-7 PMC6909516

[44] Houben CHM Spruit MA Groenen MTJ , et al. Efficacy of advance care planning: a systematic review and meta-analysis. J Am Med Dir Assoc 2014; 15(7): 477–489. 24598477 10.1016/j.jamda.2014.01.008

[45] Dixon J Matosevic T Knapp M. The economic evidence for advance care planning: Systematic review of evidence. Palliat Med 2015; 29(10): 869–884. 26060176 10.1177/0269216315586659

[46] Sanghera S Coast J. Measuring quality-adjusted life-years when health fluctuates. Value Health 2020; 23(3): 343–350. 32197730 10.1016/j.jval.2019.09.2753

[47] Wilkinson A Slatyer S McCullough K , et al. Exploring the quality of life at the end of life (QUAL-E) instrument with Australian palliative care hospital patients: hurdles and directions. J Palliat Care 2014; 30(1): 16–23. 24826439

[48] National Guideline Centre (UK). End of life care for adults: service delivery. National Guideline Centre (UK). London: Institute for Health and Care Excellence, 2019. 31633897

[49] Connor SR , et al. Global Atlas of Palliative Care at the end of Life. London: W.P.C. Alliance and W.H. Organization, 2014.

[50] Coast J Bailey C Canaway A , et al. “It is not a scientific number it is just a feeling”: populating a multi-dimensional end-of-life decision framework using deliberative methods. Health Econ 2021; 30(5): 1033–1049. 33647181 10.1002/hec.4239 PMC8129721

